# Don’t Drown with a Shingle within Reach

**Published:** 1881-10

**Authors:** 


### DON'T DROWN WITH A SHINGLE WITHIN REACH.

WE have seen a small boy, who could not swim a stroke, propel himself back and forth across a deep, wide pond, by means of a board that would not sustain five pounds weight. In fact, that sometime small boy is now writing this. Children and all others should have practice in the sustaining power of water. In nine cases out of ten, the knowledge that what will sustain a pound weight is all that is necessary to keep one's head above water, will serve better in emergencies than the greatest expertness as a swimmer. A person unfamiliar with the buoyant power of water will naturally try to climb on top of the floating object on which he tries to save himself. If it is large enough, that is all right. But it is generally not large enough, and half of a struggling group is often drowned in the desperate scramble of a life-and-death struggle to climb on top of a piece of wreck or other floating object, not large enough to keep them all entirely above water. This often happens when pleasure boats capsize. All immediately want to get out of the water on top of the overturned half-filled boat, and all are drowned except those whom the wrecked craft will wholly bear up. If they would simply trust the water to sustain ninety-nine hundredths of the weight of their bodies, and the disabled boat the other hundredth, they might all be saved under most circumstances. An overturned, or water-filled wooden boat will sustain more people in this way than it will carry. It would keep the heads above water of as many people as could get their hands on the gunwale. These are simple facts, easily learned, and may some day save your life.

In 1864, Capt. Ned. Wakeman undertook to run a North River steamer from New York to San Francisco *via* Cape Horn. He had a crew of about twenty men. When off Cape Hatteras, the natural swell of the sea soon broke the steamboat in pieces. They had but one small boat, that would not have carried half the crew. Capt. Wakeman armed himself with a club and took a seat in this boat. He then directed his men to leave the wreck and remain in the water while reaching up and taking hold of the gunwales of the small boat that he occupied; but he would only permit each one to bear on the gunwales hard enough to keep his mouth above the water, and he threatened to club any man who should disobey his orders; they knew him well enough to know that he would keep his word. In this way he kept his crew from drowning for fifteen hours, and when rescued by a passing vessel the little boat was more than half full of water, and would probably have sunk under the weight of three men had they been in the boat. It may be well to mention an incident connected with this disaster and rescue. The chief engineer was a young man named Frank Galvin. I had been acquainted with him for years. He was a very nervous and a very timid man, and his lack of coolness and self-possession cost him his life. When the rest of the crew were in the water and hanging on to the gunwales of the small boat, Galvin was seen standing on the stern of the steamer with his arms folded, looking into the sea. They paddled the small boat to within a few feet of him and urged him to leave the wreck, but he made no reply to all their entreaties but seemed dazed, and stood like a statue, and went down with the wreck without uttering a sound. In all emergencies there is safety only in coolness and determination. Thousands of lives are every year lost for want of these.

